# A retrospective analysis of venous thromboembolism trends in chemotherapy‐induced anemia: Red blood cell transfusion versus erythrocyte stimulating agent administration

**DOI:** 10.1002/jha2.18

**Published:** 2020-05-26

**Authors:** Emily J. Bryer, Michael J. Kallan, Ting‐Shan Chiu, Katharina M. Scheuba, David H. Henry

**Affiliations:** ^1^ Pennsylvania Hospital University of Pennsylvania Health System Philadelphia Pennsylvania USA; ^2^ Department of Biostatistics Epidemiology, and Informatics Perelman School of Medicine University of Pennsylvania Philadelphia Pennsylvania USA; ^3^ Data Analytics Center Perelman School of Medicine University of Pennsylvania Philadelphia Pennsylvania USA

**Keywords:** anemia, chemotherapy, erythropoietin, thrombosis, transfusion

## Abstract

**Background:**

Patients receiving a variety of chemotherapy regimens often develop chemotherapy‐induced anemia (CIA), which contributes to poor outcomes including increased mortality. Prompt and effective treatment of CIA is essential to prevent fewer chemotherapy dose delays and reductions. Optimal therapy of CIA is controversial and involves the solitary and combined use of intravenous iron, red blood cell (RBC) transfusions, and erythropoietin stimulating agents (ESAs). Despite the baseline coagulopathies present in patients with malignancy, administration of both RBC transfusions and ESAs is associated with venous thromboembolism (VTE). It remains unknown whether the risk of VTE in patients with CIA is greater among patients who receive RBC transfusions or ESAs.

**Methods:**

A retrospective study analyzed 10,269 University of Pennsylvania Health System patients with malignancies of various type, stage, and histopathology who developed CIA between 2008 and 2017. Using multivariate Cox regression, we determined adjusted hazard ratios (and corresponding 95% confidence intervals) of VTE development after adjusting for RBC and ESA intervention (all during the 90 days following CIA diagnosis).

**Results:**

Among the 10,269 patients with CIA, 2,642 (25.7%) developed a VTE within the 90‐day period. VTE risk following RBC transfusion (HR = 1.37, 95% CI 1.24‐1.50, *P* < .001) was more than twice as common as VTE risk following ESA administration (HR = 0.53, 95% CI 0.40‐0.69, *P* < .001).

**Conclusion:**

While both RBC transfusion and ESA are independently associated with VTE, our data suggest a greater risk of VTE development with RBC transfusion as compared with ESA.

## 1 INTRODUCTION

### 1.1 Malignancy‐associated thrombosis

Patients with malignancy have a sevenfold increased risk of thrombosis compared to the general population [1] with an estimated incidence of 4‐20% [2]. Thromboembolism, along with infection, is a leading cause of death in oncology patients, second only to malignancy itself [3]. The association between malignancy and coagulopathy is complex. The heterogeneity of sample size, tumor burden, treatment site, and detection of VTE make the incidence challenging to precisely estimate [4]. The predilection for thrombosis is due to a variety of mechanisms including neoplastic secretion of pro‐coagulants, activation of cysteine protease, and vascular invasion [5]. Tumor type, stage, degree of local invasion, and/or metastasis as well as the use of steroids and growth stimulating factors are associated with increased incidence of thromboembolisms in patients with malignancy [6, 7, 8]. Other factors that may contribute to the development of VTE in patients with cancer include history of a VTE, inherited or acquired mutations, hypercoagulability, thrombocytosis, recent immobilization/surgery, and hypertension [9].

### 1.2 Chemotherapy‐associated thrombosis

Approximately 20% of patients with cancer experience a VTE during their therapy course [10]. Etiology of thrombosis in patients receiving chemotherapy is multifactorial and may involve endothelial cell damage with resultant platelet activation and inflammatory cytokine cascade [5]. While different chemotherapies have varying predilections for coagulopathy, cisplatin is particularly associated with a high rate of VTE with an estimated incidence of 18% [11]. In addition to chemotherapy, other oncologic therapies increase the risk of thrombosis and include hormonal therapy, anti‐angiogenic therapy, immunomodulatory drugs, ESAs, and blood transfusions [6, 12]. Khorana et al developed a “Risk Assessment Model” for the development of thrombosis in patients with CIA (2008) and estimates the risk of thrombosis through five predictive variables: (a) site of malignancy, (b) platelet count ≥ 350 000/mL, (c) hemoglobin level < 10 g/dL or the use of ESA, (d) leukocyte count ≥11 000/mL, and (e) body mass index ≥35 kg/m^2^. This “Risk Assessment Model” also known as “The Khorana Score” has been validated in both prospective and retrospective observational studies [11, 13, 14, 15, 16].

**SUMMARY**

- Anemia is a common and unfortunate consequence of chemotherapy
- Chemotherapy‐induced anemia (CIA) contributes to dose reductions and dose delays
- Optimal treatment of CIA is controversial and involves blood transfusions, iron, and erythropoietin stimulating agents
- Both blood transfusions and erythrocyte stimulating agents are associated with thrombosis
- This study involves a large population >10 000 people and includes all types of cancer and chemotherapy
- RBC transfusion should not be withheld from patients with symptomatic CIA since it does not carry a higher risk of VTE compared with no anemia treatment
- This study demonstrates a greater risk of venous thromboembolism in patients with CIA following RBC transfusion as compared with ESA
- Moving forward, patients with CIA may benefit from therapy with ESA compared with RBC due to a lower risk of VTE

### 1.3 RBC, ESA, and VTE in CIA

RBC transfusion is a transient therapeutic strategy for those with CIA to provide supportive care and prolong patient survival to allow chemotherapy to be effective [17]. There are many consequences of RBC transfusion that include immunomodulatory effects, pathogen transmission, as well as both arterial and venous thromboses [18]. Mechanisms of RBC transfusion‐associated thrombosis include platelet and endothelial cell activation along with the transmission of pro‐coagulant and pro‐inflammatory cellular components including CD40L, plasminogen activator inhibitor 1, nitric oxide, and adenosine diphosphate [19].

ESAs are a class of recombinant medications that induce red cell proliferation via utilization of iron stores that are necessary for effective erythropoiesis [10]. While this class of medications was initially used clinically in renal dialysis patients in the 1980s [20], its effects on boosting erythropoiesis have been applied to the fields of hematology and oncology and are used in conjunction with myelotoxic chemotherapies to supplement erythrocyte proliferation and maturation. ESAs such as recombinant erythropoietin and darbepoetin alfa are used to reduce CIA and fatigue that result from myelotoxic therapies [21]. Administration of ESA in CIA is associated with fewer RBC transfusions, improved quality of life [22] as well as improved mood and cognitive function in cancer patients receiving chemotherapy [23].

While ESA and RBC transfusion are treatment modalities used in CIA, both are associated with thrombotic diatheses [18, 24, 25, 26, 27, 28, 29]. The associations between VTE, ESA, and RBC transfusions are both complex and poorly understood. Some data suggests that the degree by which RBC transfusion influences the risk of VTE is dependent upon the thrombotic risk associated with a specific malignancy [30].

Indications for ESA use have changed over the past two decades, especially following recognition of increased incidence of thrombosis in patients with CIA who receive ESA to achieve higher target hemoglobin levels [27]. In March 2007, the FDA issued a black box warning on ESAs to use the lowest dose possible to achieve a hemoglobin level high enough to avoid blood transfusions to avoid both venous and arterial thromboemboli (Information for Healthcare Professionals: Erythropoiesis Stimulating Agents (ESA), Aranesp (darbepoetin), Epogen (epoetin alfa), and Procrit (epoetin alfa). FDA Alert, March 2007.) As a result of this black box warning, administration patterns of ESA in CIA have changed.

The American Society of Clinical Oncology and American Society of Hematology 2019 Clinical Practice Guideline Update for epoetin and darbepoetin in adult patients receiving myelotoxic chemotherapy with a hemoglobin < 10 g/dL recommend consideration of both risks and benefits in patients for whom ESAs are prescribed [31]. Current data regarding ESA use and thrombosis is limited as many of the available studies were conducted prior to 2007 and therefore used higher target hemoglobin thresholds; as a result, the reported incidence of thromboses is confounded by the conditions under which ESA was administered. Additionally, prior data regarding the measurement of ESA use and blood transfusion in patients with malignancy included both patients with cancer‐related anemia as well as those with CIA, posing a challenge to assess VTE in solely CIA. This study aims to compare the risk of VTE in patients with CIA who receive RBC transfusion and patients with CIA who receive ESA.

## 2 METHODS

A retrospective analysis was performed of patients within the University of Pennsylvania Health System from 2008 to 2017 in accordance with Institutional Review Board standards. Four hospitals were included: Hospital of the University of Pennsylvania, Pennsylvania Hospital, Presbyterian Medical Center, and Chester County Hospital. The data included both inpatients and outpatients who had an ICD9 or ICD10 diagnosis of CIA as defined by The National Cancer Institute Anemia Scale (from NCCN):

- Grade 0 = Normal limits = hemoglobin 12–16 g/dL for women and 14–18 g/dL for men
- Grade 1 = Mild = 10 g/dL through lower limit of normal
- Grade 2 = Moderate = 8–10 g/dL
- Grade 3 = Severe = 6.5‐8 g/dL
- Grade 4 = Life threatening ≤ 6.5 g/dL
- Grade 5 = Death

Within the category of CIA, patients were subdivided by receipt of either red blood cell (RBC) transfusion or/and erythrocyte stimulating agent (ESA) as defined by darbepoetin or erythropoietin. Data were further analyzed by the frequency and timing of the development of a venous thromboembolism (VTE) within 90 days following administration of RBC or ESA. Thromboembolic events were defined as either deep venous thrombosis (DVT) or pulmonary embolus. Diagnostic methods of VTE included compression ultrasound, computed tomographic pulmonary angiogram, and ventilation/perfusion scintigraphy (V/Q) scan. Isolated elevated d‐dimer without evidence on imaging studies did not qualify as a diagnosed VTE. Other variables examined included age, gender, hemoglobin, platelet, and INR values at the time of clot as well as stage, primary organ, and histopathology of malignancy. Following electronic extraction of this data set, some data regarding malignancy stage, primary organ of malignancy, and histopathology was unknown. To supplement this data, review of individual electronic medical charts was conducted. Patients who had diagnoses of more than one malignancy where analyzed by the type of malignancy that they were undergoing chemotherapy for at the time of CIA diagnosis. Patients undergoing chemotherapy with multiple concurrent tumors (eg, Li‐Fraumeni syndrome) were distinguished as a sub‐group. Cancers without a clear primary site of origin were designated as “unknown.”

Exclusionary criteria as defined by ICD9 and ICD10 codes included patients who were actively using tobacco as well as patients who received any other type of blood product aside from red blood cells such as whole blood, platelets, fresh frozen plasma, and cryoprecipitate. Patients were also excluded if they had an inferior vena cava filter at the time of VTE diagnosis, had a previously diagnosed coagulopathy (including Protein C and S deficiency, Factor V Leiden, antiphospholipid antibody syndrome, hyperhomocysteinemia), as well as patients on therapeutic anticoagulation at the time of the clot diagnosis (including warfarin, heparin, rivaroxaban, apixaban, edoxaban, and pradaxa.) Patients were excluded if they developed CIA and did not have a malignancy; this included patients receiving chemotherapy agents for immunosuppression of systemic inflammatory disease states (systemic lupus erythematosus, inflammatory bowel disease, amyloidosis, giant cell arteritis, microscopic polyangiitis) and organ transplant recipients (lung, heart, liver, pancreas, kidney).

Using multivariate Cox regression, we determined adjusted hazard ratios (and corresponding 95% confidence intervals) of VTE development after adjusting for RBC and ESA intervention (all during the 90 days following CIA diagnosis). Data were then stratified by gender (men and women) and age (65+ and <65) for further analysis of VTE trends. Further analyses were conducted according to organ of origin: lung, soft tissue, gastric (stomach, small bowel, pancreas), colorectal (colon, anus, rectum), biliary (intrahepatic, extrahepatic, gallbladder), breast, bone marrow (lymphoma, leukemia, multiple myeloma, monoclonal gammopathy of unknown significance, myelofibrosis, myelodysplasia), head and neck (larynx, oral cavity, tongue, pharynx, oropharynx, lip, salivary glands, thymus, thyroid), and genitourinary (prostate, kidneys, bladder, testicular, ovaries, uterus, ureter). Results were further stratified by the most common types of histopathology within the sample. Hemoglobin and platelet values were extracted at the time of diagnosis of CIA and VTE as well as prior to RBC transfusion and ESA administration.

## 3 RESULTS

Among the 10,269 patients with CIA, 7878 patients (76.7%) did not receive any therapy during the 90‐day period for their anemia (neither RBC nor ESA), 2008 patients received RBC transfusion, 246 received ESA, and 137 patients received both (Table 1). Among all patients, 2642 (25.7%) developed a VTE within the 90‐day period. VTE risk following RBC transfusion (HR = 1.37, 95% CI 1.24‐1.50, *P* < .001) was more than twice the risk following ESA administration (HR = 0.53, 95% CI 0.40‐0.69, *P* < .001). Within the patient population who received both RBC and ESA, 12/137 (8.8%) developed a VTE.

**TABLE 1:** Treatment and outcomes within 90 days following CIA diagnosis

	No Anemia Treatment	RBC only	ESA only	RBC and ESA	Total Patients
Patients with VTE / (All patients)	2043 / (7878) = 25.9%	545 / (2008) = 27.1%	42 / (246) = 17.1%	12 / (137) = 8.8%	2642 / (10 269) = 25.7%
		HR = 1.37, 95% CI 1.24‐1.50, *P* < .001	HR = 0.53, 95% CI 0.40‐0.69, *P* < .001		
Men with VTE /(All male patients)	1056 / (3898) = 27.1%	276 / (1044) = 26.4%	17 / (94) = 18.1%	3 / (64) = 4.7%	1352 /(5100) = 26.5%
		HR = 1.24, 95% CI 1.09‐1.41, *P* = .001	HR = 0.47, 95% CI 0.30‐0.73, *P* = .001		
Women with VTE / (All female patients)	987 / (3979) = 24.8%	269 / (964) = 27.9%	25 / (152) = 16.4%	9/ (73) = 12.3%	1290 / (5168) = 25.0%
		HR = 1.51, 95% CI 1.32‐1.72, *P* < .001	HR = 0.58, 95% CI 0.41‐0.81, *P* = .002		
Age < 65 with VTE / (All patients < 65)	900 / (3335) = 27.0%	238 / (907) = 26.2%	15 / (72) = 20.8%	4 / (51) = 7.8%	1157 / (4365) = 26.5%
		HR = 1.26, 95% CI 1.09‐1.45, *P* = .001	HR = 0.57, 95% CI 0.36‐0.89, *P* = .015		
Age 65+ with VTE / (All patients 65+)	1143 / (4543) = 25.2%	307 / (1101) = 27.9%	27 / (174) = 15.5%	8 / (86) = 9.3%	1485 / (5904) = 25.2%
		HR = 1.45, 95% CI 1.28‐1.64, *P* < .001	HR = 0.51, 95% CI 0.37‐0.72, *P* < .001		

Note: In the above table, each value listed as (x / y), x pertains to all patients who received the specified treatment who had a VTE, while y pertains to all patients who received the specified treatment.

Platelet and hemoglobin values were analyzed at the time of CIA diagnosis, VTE diagnosis, and prior to both RBC transfusion and ESA administration (Table 4). The mean hemoglobin at the diagnosis of CIA was 9.8 with a standard deviation of 1.7 was similar to the mean hemoglobin at the diagnosis of VTE of 9.2 with a SD of 1.5. The median hemoglobin prior to RBC transfusion was 8.8 with a range of 2.8‐15.3 that is slightly lower than the median hemoglobin prior to ESA administration of 9.2 with a range of 3.7‐13.0. The median platelet value at the time of CIA diagnosis was 161.0, slightly higher than the median platelet value at the time of VTE diagnosis 142.0.

Among patients <65 years of age, VTE was approximately twice as likely among patients who received RBC (HR = 1.26, 95% CI 1.09‐1.45, *P* = .001) compared with those who received ESA (HR = 0.57, 95% CI 0.36‐0.89, *P* = .015; Table 1). Among patients 65 years old and older, those who received RBC (HR = 1.45, 95% CI 1.28‐1.64, *P* < .001) had approximately three times the risk of VTE compared with those who received ESA (HR = 0.51, 95% CI 0.37‐0.72, *P* < .001). Following RBC transfusion, VTE risk for men (HR = 1.24, 95% CI 1.09‐1.41, *P* = .001) and women (HR = 1.51, 95% CI 1.32‐1.72, *P* < .001) was approximately three‐fold the VTE risk following ESA administration for men (HR = 0.47, 95% CI 0.30‐0.73, *P* = .001) and women (HR = 0.58, 95% CI 0.41‐0.81, *P* = .002), respectively (Table 1).

Analyses of VTE trend by primary organ (Table 2) yielded a statistically significant increase in VTE following RBC transfusion in patients with colorectal cancer (HR = 2.40, 95% CI 1.21‐4.75, *P* = .012), cancer of the bone marrow (HR = 1.36, 95% CI 1.12‐1.66, *P* = .002), and head and neck cancer (HR = 2.47, 95% CI 1.27‐4.80, *P* = .008). Patients with soft tissue malignancies who received RBC transfusion actually had a lower rate of VTE (HR = 0.40, 95% CI 0.25‐0.63, *P* < .001). Following RBC transfusion, patients with sarcoma demonstrated a lower rate of VTE (HR = 0.67, 95% CI 0.47‐0.96, *P* = .031).

**TABLE 2:** VTE in CIA by primary malignancy

	No Treatment	RBC only	ESA only	RBC and ESA	Total Patients
Lung	164/ (471) = 34.8%	35 /(97) = 36.1%	8 / (28) = 28.6%	1 /(16) = 6.3%	208 /(612) = 34.0%
		HR = 1.19, 95% CI 0.83‐1.71, *P* = .34	HR = 0.52, 95% CI 0.26‐1.01, *P* = .34		
Soft Tissue	126 /(185) = 68.1%	18 / (46) = 39.1%	10 /(14) = 71.4%	4 / (18) = 22.2%	158/(263) = 60.1%
		HR = 0.40, 95% CI 0.25‐0.63, *P* < .001	HR = 0.73, 95% CI 0.42‐1.27, *P* = .26		
Colon	48 /(233) = 20.6%	9 /(29) = 31.0%	0 / (9) = 0.0%	1 /(3) = 33.3%	58/(274) = 21.2%
		HR = 2.40, 95% CI 1.21‐4.75, *P* = .012	HR = 0.38, 95% CI 0.05‐2.72, *P* = .33		
Gastric	51 /(207) = 24.6%	13 /(47) = 27.7%	1 /(11) = 9.1%	0 /(1) = 0.0%	65 /(266) = 24.4%
		HR = 1.78, 95% CI 0.97‐3.28, *P* = .06	HR = 0.36, 95% CI 0.05‐2.58, *P* = .31		
Biliary	7 / (45) = 15.6%	1 / (6) = 16.7%	2 /(4) = 50.0%	0 /(1) = 0.0%	10 / (56) = 17.9%
		HR = 1.11, 95% CI 0.14‐8.78, *P* = .92	HR = 4.58, 95% CI 0.97‐21.61		
Breast	64 /(347) = 18.4%	6 /(36) = 16.7%	1 /(14) = 7.1%	1 / (2) = 50.0%	72/(399) = 18.0%
		HR = 1.41, 95% CI 0.65‐3.07, *P* = .39	HR = 0.67, 95% CI 0.16‐2.72, *P* = .57		
Bone Marrow	367 /(887) = 41.4%	136 / (311) = 43.7%	8 / (21) = 38.1%	1 /(11) = 9.1%	512/(1230) = 41.6%
		HR = 1.36, 95% CI 1.12‐1.66, *P* = .002	HR = 0.65, 95% CI 0.34‐1.26, *P* = .20		
Head and Neck	33 /(213) = 15.5%	12 /(39) = 30.8%	2 /(4) = 50.0%	0/ (2) = 0.0%	47/(258) = 18.2%
		HR = 2.47, 95% CI 1.27‐4.80, *P* = .008	HR = 2.75, 95% CI 0.66‐11.54, *P* = .17		
Genitourinary	50 /(157) = 31.8%	10 /(23) = 43.5%	2 / (3) = 66.7%	1 /(3) = 33.3%	63/(186) = 33.9%
		HR = 1.80, 95% CI 0.93‐3.49, *P* = .08	HR = 1.30, 95% CI 0.40‐4.26, *P* = .66		

Note: In the above table, each value listed as (x/y), x pertains to all patients who received the specified treatment who had a VTE, while y pertains to all patients who received the specified treatment.

When rates of VTE following RBC transfusion were further stratified by histopathology (Table 3), patients with lymphoma (HR = 2.24, 95% CI 1.60‐3.12, *P* < .001) and carcinoma (HR = 1.97, 95% CI 1.49‐2.60, *P* < .001) had increased risks of VTE. There was no statistically significant difference in VTE rate following RBC transfusion among patients with leukemia or multiple myeloma. Carcinoma was the only type of malignancy with a statistically significant differences between rates of VTE following ESA administration (HR = 0.58, 95% CI 0.35‐0.94, *P* = .029).

**TABLE 3:** VTE in CIA by Histopathology of Malignancy

	No Treatment	RBC only	ESA only	RBC and ESA	Total Patients
Lymphoma	126 / (346) = 36.4%	49 /(95) = 51.6%	4 / (7) = 57.1%	0 /(5) = 0.0%	179/(453) = 39.5%
		HR = 2.24, 95% CI 1.60‐3.12, *P* < .001	HR = 0.60, 95% CI 0.22‐1.64, *P* = .32		
Leukemia	90 /(182) **= **49.5%	36 / (96) = 37.5%	2 / (3) = 66.7%	0 /(1) = 0.0%	128/(282) = 45.4%
		HR = 0.79, 95% CI 0.53‐1.15, *P* = .22	HR = 1.55, 95% CI 0.38‐6.29, *P* = .54		
Carcinoma	301 /(1091) = 27.6%	57 / (147) = 38.8%	14 / (62) = 22.6%	3 /(27) = 11.1%	375/(1327) = 28.3%
		HR = 1.97, 95% CI 1.49‐2.60, *P* < .001	HR = 0.58, 95% CI 0.35‐0.94, *P* = .029		
Sarcoma	159 / (251) **= **63.3%	33 /(69) = 47.8%	11 /(16) = 68.8%	5 /(21) = 23.8%	208/(357) = 58.3%
		HR = 0.67, 95% CI 0.47‐0.96, *P* = .031	HR = 0.69, 95% CI 0.41‐1.16, *P* = .16		
Multiple Myeloma	89 / (204) = 43.6%	28 / (57) = 49.1%	1 / (11) = 9.1%	1 /(2) = 50%	119/(274) = 43.4%
		HR 1.58, 95% CI 1.04‐2.41, *P* = .032	HR = 0.36, 95% CI 0.09‐1.44, *P* = .15		

Note: In the above table, each value listed as (x/y), x pertains to all patients who received the specified treatment who had a VTE, while y pertains to all patients who received the specified treatment.

**TABLE 4:** Hemoglobin and Platelet Values

	Diagnosis of CIA	Diagnosis of VTE	Prior to RBC Transfusion	Prior to ESA Administration
Hemoglobin	n = 9,097 (of 10 269 total):	n = 2434 (of 2642 total):	n = 1966 (of 2008 total):	n = 215 (of 246 total)
	mean = 9.8 (SD = 1.7)	mean = 9.2 (SD = 1.5)	mean = 8.9 (SD = 1.6)	mean = 9.2 (SD = 1.1)
	median = 9.6	median = 9.2	median = 8.8	median = 9.2
	range = (2.8‐17.9)	range = (3.1‐15.1)	range = (2.8‐15.3)	range = (3.7‐13.0)
	IQR = (8.7‐10.8)	IQR = (8.3‐10.1)	IQR = (7.9‐9.9)	IQR = (8.5‐9.9)
Platelets	n = 4520 (of 10,269 total):	n = 997 (of 2642 total):		
	Mean = 182.0 (SD = 135.4)	Mean = 173.4 (SD = 142.9)		
	median = 161.0	median = 142.0		
	range = (3‐1283)	range = (3‐1283)		
	IQR = (81‐251)	IQR = (69‐243)		

Abbreviations: n, number; SD, standard deviation.

**FIGURE 1:**
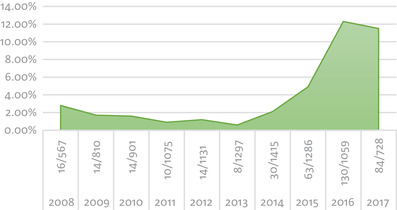
Annual ESA use

**FIGURE 2:**
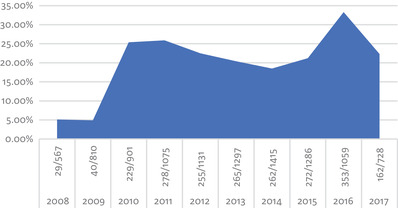
Annual RBC use

Investigation of each product (RBC, ESA, and no anemia treatment) relative to the other two products by annual percentage demonstrated an initial decrease of ESA use from 2008 to 2013 followed by a sharp increase through 2017 (Figure 1). RBC use appears to have been among the 4‐5% range from 2008 to 2009 followed by an abrupt increase to approximately 20% that has been maintained through 2017 (Figure 2).

## 4 DISCUSSION

Although RBC transfusions and ESA are both thrombogenic, the duration of time that the corresponding hyper‐coagulable state persists is yet to be defined. An inclusion period of 90 days following RBC or ESA administration was arbitrarily selected in an effort to isolate an associated VTE. Among patients with CIA, the analysis of VTE trends among patient who receive RBC versus ESA have not yet been studied. Our data suggest that patients with CIA who receive RBC transfusion have more than twice the likelihood of developing a VTE compared with those who receive ESA. The data demonstrate that the risk of VTE following RBC transfusion in patients with CIA <65 years old is more than twice as high as those patients <65 years old who receive ESA. Interestingly, despite the pro‐thrombotic nature of estrogen, gender had no statistically significant effect on VTE in CIA.

Hemoglobin values at the time of diagnosis of both CIA and VTE were similar, with interquartile ranges of 8.7‐10.8 g/dL and 8.3‐10.1 g/dL, respectively. Prior to treatment of CIA with either RBC or ESA, interquartile ranges of hemoglobin values from the date closest to CIA diagnosis were similar at 7.9‐9.9 g/dL and 8.5‐9.9 g/dL, respectively. Platelet values at the time of diagnosis of CIA (interquartile range 81‐251) were only slightly higher than platelet values at the time of diagnosis of VTE (interquartile ranges 69‐243).

Among patients with soft tissue malignancies, administration of RBC transfusion was associated with a lower rate of VTE (HR = 0.40, 95% CI 0.25‐0.63, *P* < .001) compared with those with soft tissue malignancies who did not receive RBC transfusion. Patients with colorectal cancer who received RBC transfusion demonstrated a greater than twofold increase in rate of VTE (HR = 2.40, 95% CI 1.21‐4.75, *P* = .012) that was similar to the rate of VTE among patients with head and neck cancer following RBC transfusion (HR = 2.47, 95% CI 1.27‐4.80, *P* = .008). Following RBC transfusions, patients with cancer of the bone marrow were associated with higher rates of VTE (HR = 1.36, 95% CI 1.12‐1.66, *P* = .002). It remains unclear whether the aforementioned statistically significant differences between VTE rate following RBC transfusion among patients stratified by location of malignancy are related to disease pathology, chemotherapy, or whether or not this small subset of patients is representative of the full sample size and general population of patients with CIA.

VTE is common in patients with lymphoma [18]. Three risk factors associated with VTE in patients with lymphoma include poor performance status, CNS localization, and tumor bulk > 10 cm [32]. This study demonstrates that among patients with lymphoma and CIA, transfusion of RBC further escalates the risk of VTE twofold compared to patients with lymphoma who do not receive RBC transfusions. Carcinoma was the only type of histopathology included in the study that exemplified statistically significant rates of VTE following both ESA administration and RBC transfusion. Remarkably, administration of RBC resulted in a twofold increase of VTE compared with an approximately 50% decrease of VTE rates following ESA administration in patients with carcinoma. Stratification of patients yielded sarcoma as the sole histopathology associated with decreased rates of VTE following RBC transfusion (HR = 0.67, 95% CI 0.47‐0.96, *P* = .031). While the data regarding VTE in patients with sarcoma is sparse, some additional factors associated with decreased risk of VTE in those with soft tissue sarcoma include central venous catheterization, high‐risk surgery, and surgery of primary; since the three aforementioned events all occur in the hospital, this was hypothesized to be due to the provision of VTE prophylaxis in the inpatient setting [33].

While many malignancies are associated with VTE, some are more closely correlated than others. Patients with multiple myeloma a ninefold risk of VTE compared with the general population [34]. In addition to the inherent coagulopathy of multiple myeloma, our data suggest that RBC transfusion further augments thrombosis (HR = 1.58, 95% CI 1.04‐2.41, *P* = .032). Some other risk factors for thrombosis in patients with multiple myeloma include age, body mass index, and VTE prior to diagnosis of multiple myeloma, and thalidomide [34].

Our data is not the first to suggest that patients with cancer and anemia have a higher rate of VTE following RBC transfusion compared with ESA administration [30]. Although the aforementioned data include patients with anemia that is not necessarily chemotherapy‐induced, the higher risk for VTE following RBC transfusions compared with ESA persists despite adjusting for a variety of risk factors and using a restrictive definition of VTE [30]. Hypotheses as to why patients with CIA may have a higher risk of VTE development with RBC transfusions compared with ESA may involve the inflammatory response and reaction to cytokines following receipt of an exogenous product (RBC) rather than the provision of a stimulus (ESA) to generate endogenous red blood cells. The rapidity of hemoglobin change following RBC may account for an inflammatory and prothrombotic state compared with the gradual increase in hemoglobin weeks following ESA administration. Furthermore, blood storage time may impact the rate of VTE. The oldest blood products (within the 42‐day shelf life) are typically transfused first, which may result in the transmission of higher numbers of cytokines and an associated exaggerated inflammatory response that may instigate a thrombosis.

ESA is commonly administered one of two ways: a once‐weekly high dose or daily for a week with lower doses. The juxtaposition of VTE rates between these two dose regimens has yet to be studied in CIA. Interestingly, patients with chronic kidney disease who require high‐dose erythropoietin were found to have higher levels of circulating pro‐inflammatory biomarkers IL6 and CRP that may translate to higher levels of VTE [35]. The 2007 FDA issued black box warning regarding use of epoetin alfa at higher hemoglobin levels and the corresponding risk of thrombosis following ESA administration affected clinician use of ESA. Following this warning, the use of ESA fell out of favor with fear of a poor safety profile and predisposition to VTE. As ESA use decreased following this FDA warning [36], so did RBC transfusions, peaking in 2008 [37]. Our data suggest an initial decrease in ESA use from 2008 to 2013, likely a reflection of the FDA warning, followed by an increase in ESA use from 2014 to 2017 (Figure 1). In 2015, RBC transfusions decreased by 24.4% compared to levels in 2008 [38, 39]. While national trends demonstrate a decrease in RBC transfusions, our data show an increase in RBC transfusions from 5% noted in 2008 and 2009 up through the peak of 33.3% in 2016 (Figure 2). In the midst of a shift toward a more restrictive transfusion policy, it is unclear if the discrepancy between national trends and the transfusion rates in this study are influenced by clinician preference of a higher hemoglobin target.

### 4.1 Study limitations

The study did not stratify inpatients from outpatients, the former that may confound VTE rate with likely higher rates of immobility. Unless identified incidentally, this study included only symptomatic VTE and there was no differentiation between catheter‐related thromboses and non‐catheter related thromboses. It is possible that a patient had an undiagnosed VTE prior to RBC or ESA transfusion that was subsequently identified after an attributed to the therapy. Study limitations also include the variation and subjectivity of clinician tendency to initiate treatment for CIA as well as to select the type of therapy (RBC or ESA). Despite individual review of over 10 000 charts, some data regarding type of malignancy and histopathology remained unavailable that was a major limitation of the sub‐group analyses by cancer organ of origin and histopathology, both of which were available for only about 30% of the patient population. Similarly, there was a lack of consistent data regarding cancer stage, hemoglobin, platelets, and INR at both the time of RBC or ESA administration and the time of VTE diagnosis. Although this is a retrospective study without the ability to randomize study groups, it should be noted that the available data included more RBC transfusions than ESA administrations.

## 5 FUTURE DIRECTIONS

Further investigations are needed within CIA therapy and VTE in malignancy to best identify the patients most at risk for VTE and potentially pre‐disposing factors. **“**Thrombosis is a potentially avoidable source of morbidity and mortality in ALL patients, particularly during the early phases of chemotherapy” [40, 41, 42]. Future analyses of VTE rates in patients with CIA following RBC transfusion and ESA administration may consider Khorana score, chemotherapy regimen and schedule, INR, tumor estrogen‐receptor positivity, and stage of malignancy at the time of VTE.

## 6 CONCLUSION

Thrombosis is a major source of morbidity and mortality among patients with CIA. RBC transfusion should not be withheld from patients with symptomatic CIA since it does not carry a higher risk of VTE compared with no anemia treatment. This study demonstrates an approximately twofold increase of VTE following RBC transfusion compared with the rate of VTE following ESA administration regardless of age or gender. In recognition of this hypercoagulability associated with RBC transfusions in patients with CIA, clinicians might consider the Khorana score when selecting a CIA therapy. There may be a greater role for ESA in CIA among patients with higher Khorana scores to lessen the risk of thrombosis with RBC. In addition to the thrombogenicity of the patient, clinicians may also consider the rapidity needed to improve hemoglobin with RBCs resulting in much faster correction of symptomatic anemia. In the appropriate clinical setting, patients with asymptomatic CIA who are not bleeding may benefit from therapy with ESA with a lower risk of thrombosis compared with RBC transfusion. Further research is needed to clarify the relationship between VTE among patients with CIA in an effort to decrease morbidity and mortality among patients with malignancy.

## 7 DECLARATIONS

- Ethics approval and consent to participate
  - Approved by IRB at University of Pennsylvania
- Consent for publication: all patients kept anonymously without identifying information
- Availability of data and material
  - Data and materials available per electronic medical record review
  - Data subject to third party restrictions. The data that support the findings of this study are available by request from the University of Pennsylvania Institutional Research Board. Restrictions apply to the availability of these data, which were used under license for this study.

## AUTHOR CONTRIBUTIONS

Emily Bryer and David Henry are the authors of the manuscript and designed the study. Ting‐Shan Chiu collected the data, Katharina Scheuba helped to analyze the data, and Michael Kallan performed the statistical analysis.
